# Outbreak of Fatal Piglet Diarrhea Caused by *Chromobacterium haemolyticum* in China

**DOI:** 10.1155/2023/6694913

**Published:** 2023-02-27

**Authors:** Leyi Zhang, Pengfei Zhang, Ge Xu, Kevin Sun Song, Tairun Liang, Jiawei Peng, Shoutang Wu, Ningjia Yang, Lin Wang, Yanling Liu, Xinming Zhang, Pengshuai Liang, Zheng Xu, Changxu Song

**Affiliations:** ^1^College of Animal Science & National Engineering Center for Swine Breeding Industry, South China Agricultural University, Guangzhou 510642, China; ^2^Guangdong Laboratory for Lingnan Modern Agriculture, College of Animal Science, South China Agricultural University, Guangzhou 510642, China; ^3^Lab of Respiratory Pathogens and Immunity, Department of Basic Research, Guangzhou Laboratory, Guangzhou 510320, Guangdong, China; ^4^Department of Biology, Emory University, Atlanta, GA 30322, USA

## Abstract

*Chromobacterium haemolyticum* is a fatal Gram-negative bacterium which could infect human beings. Our investigation found severe piglet diarrhea in one farm; the morbidity rate of piglets was 30.65%. Then, we isolated a nonpigmented, *β*-hemolytic Gram-negative bacillus from the clinical samples of this farm, which was designated GDHYZ30 strain. The 16S rRNA gene sequence indicated it was most closely related to *Chromobacterium haemolyticum*. Similar clinical symptoms were successfully reproduced in experimental piglets with this isolate, and the isolate was also subjected to whole genome-sequencing. It is worth noting that this *Chromobacterium haemolyticum* has been isolated from several other pig farms with diarrhea of unknown causes. To our knowledge, this is the first report that discusses *Chromobacterium haemolyticum* as a new pathogen causing diarrhea and death in piglets and transmitting through water sources, and it provides a reference for the prevention and control of human/animal infections as well as food safety.

## 1. Introduction

The genus *Chromobacterium*, consisting of seven recognized species, is a Gram-negative, rod-shaped bacterium, and its most famous specie is *Chromobacterium violaceum* with a red pigment [1]. *Chromobacterium* is mainly distributed in tropical and subtropical regions, and it is frequently isolated in water sources and humid areas [2]. *Chromobacterium haemolyticum* (*C*. *haemolyticum*) was considered as a separate species, lacking violet pigmentation but demonstrating strong hemolysis on sheep blood agar plates [3]. *C*. *haemolyticum* has been isolated from a variety of water environments, such as rice roots, lakes, rivers, and sewage effluent [4–6]. There is evidence that *C*. *haemolyticum* is mainly transmitted through water sources, and some reports suggest that *C*. *haemolyticum* can cause pneumonia and sepsis in humans, and the patients in these cases have a history of wound exposure or contact with water sources, indicating that *C*. *haemolyticum* maybe an opportunistic pathogen [7, 8]. There also have been reports of diarrhea cases in humans that were related to *C*. *haemolyticum*, but *C*. *haemolyticum* causing diarrhea or even death in other animals has not been reported yet [9].

Piglets diarrhea is a serious disease that causes great harm to the swine industry. Due to the fact that the intestinal immunity of piglets has not fully developed yet, many pathogens are susceptible to piglets, causing acute diarrhea, vomiting, dehydration, and even death [10, 11]. Diarrhea in piglets can be devastating to pig farms if the main pathogen cannot be identified and treated accordingly. Some farms use mountain springs or river water as water sources without disinfection, which often leads to infection of some bacterial diseases. *C*. *haemolyticum* is pathogenic and usually detected in water sources; however, it has been paid less attention compared to its peers. Thus, it is meaningful to conduct in-depth research on the biological and genetic characteristics of this bacteria.

Our study focused on an unexplained case of piglet diarrhea that occurred in many pig farms in south China. After excluding some regular pathogens, *C. haemolyticum* was found both in the water source and in piglet fecal samples with diarrhea. The challenge of piglets with the isolate GDHYZ30 can cause diarrhea symptoms similar to those of the field cases. And the diarrhea of piglets had been cured after changing the drinking water source, indicating that the GDHYZ30 strain contaminating the drinking water is the agent for the piglet diarrhea and death.

## 2. Materials and Methods

### 2.1. Sample Collection and Pathogen Identification

The anal swabs from diarrheic piglets and water sources from pig farms were collected. Nucleic acids were extracted to detect common diarrhea-associated viruses; primers were referred to Supplemental file 1, and bacteria were isolated using sheep blood agar plates.

### 2.2. Antibiotic Susceptibility Assay

Antimicrobial susceptibility profiles were determined according to the CLSI (Clinical and Laboratory Standards Institute) [12]. The inhibition zone diameter was measured after 24 h incubation at 37°C.

### 2.3. Transmission Electron Microscopy

GDHYZ30 were grown for 24 h in LB medium, centrifuged (5000 g, 5 min), and resuspended in distilled water. Samples were placed on a carbon-coated grid (200 mesh) and stained with 1% phosphotungstic acid (PTA). The grid was air dried and examined in a Talos L120 C microscope (Thermo Fisher, USA).

### 2.4. Experimental Infection Trial

Nine 30-day-old healthy piglets were randomly divided into three groups. 2 mL of the 3.5 × 10^9 colony-forming unit (CFU) of GDHYZ30 were administered orally or intramuscularly injected, respectively, and 2 mL of PBS were administered orally to the control group.

### 2.5. Histological Examination

Fresh piglet intestinal tissues were collected from piglets fixed in 10% neutral buffered formalin, stained with hematoxylin and eosin, and examined under a light microscope.

### 2.6. Animal Ethics and Welfare

Animal studies were carried out in strict accordance with the recommendations made in the Guide for the Animal Care & Health and Supervisory Committee. Protocols were approved by the Committee on the Ethics of Animal Experiments of the South China Agricultural University.

## 3. Results

### 3.1. Clinical and Biological Properties

**Figure 1:**
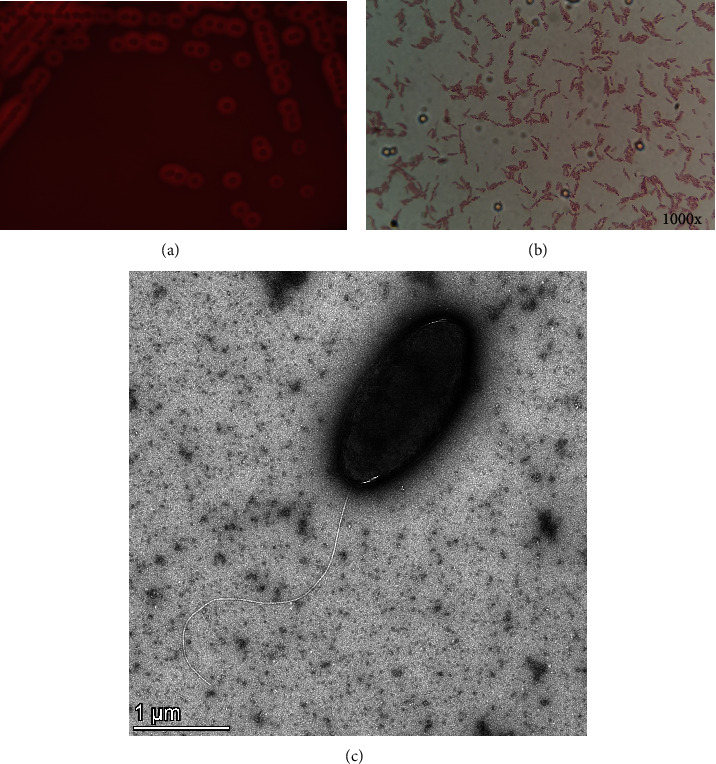
Bacterial isolation and identification. (a) *β*-hemolysis on sheep blood agar plates; (b) Gram staining; and (c) transmission electron microscopy.

Our investigation focused on severe piglet diarrhea that occurred at one farm in south China, where the morbidity rate of piglets was 30.65% (149/486) and the mortality rate was 35.57% (53/149). To figure out the pathogen responsible for diarrhea in piglets, we collected fecal samples from piglets with diarrhea and tested a variety of diarrhea-causing viruses. The viruses tested includes porcine epidemic diarrhea virus (PEDV), porcine deltacoronavirus (PDCov), transmissible gastroenteritis virus (TGEV), swine acute diarrhea syndrome coronavirus (SADS-CoV), porcine rotavirus (PRoV), porcine circovirus type 3 (PCV3), porcine circovirus-like viruses (PCLV), and other common pathogens listed in the supplemental files. The viral presence had been monitored by PCR methods, and all the above pathogens had not been detected. Excitingly, a Gram-stained negative, rod-shaped (Figure 1(b)) *β*-hemolysis bacterium was isolated on sheep blood agar plates (Figure 1(a)). The following identification verified that the isolated bacterium is about 3 *μ*m in diameter and with a single polar flagella (Figure 1(c)). The strain was purified by colonization and named the GDHYZ30 strain.

### 3.2. Genetic Evolution and Prevalence

**Figure 2:**
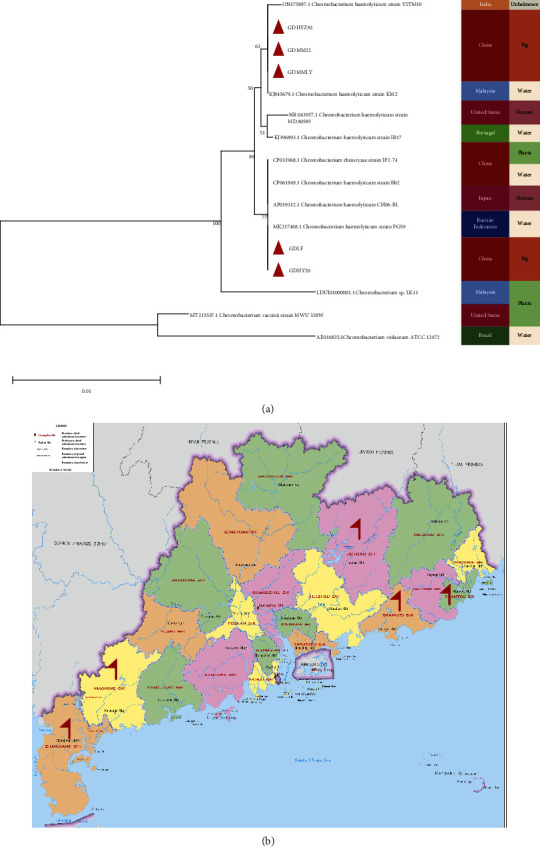
Phylogenetic relationship and epidemiological. (a) Neighbour-joining phylogenetic tree based on a comparison of the 16S rRNA gene sequences of isolated strain and its closest relative bacterium strains; and (b) regions of *Chromobacterium haemolyticum* infection in Guangdong province. The region affected with *Chromobacterium haemolyticum* was indicated in red flag.

In order to study the genetic characteristics of the bacterium, we carried out the whole gene-sequencing analysis. The 16S rRNA phylogenetic tree analysis showed that the bacterium belongs to *Chromobacterium*, which is the same branch as *C. haemolyticum.* We compared *C. haemolyticum* from different sources and found that GDHYZ30, GDMM22, and GDMMLY were closest to the KM2 strain, and GDLY and GDHY20 were closest to the PGS9 strain (Figure 2(a)). The climatic characteristics of Guangdong Province are more suitable for the reproduction of *Chromobacterium*, so we expanded the detection range. Consequently, *C. haemolyticum* was detected in water sources and an anal swab of piglets in other areas with unexplained diarrhea. The bacteria were specifically detected in five regions, including HEYUAN, MAOMING, ZHANJIANG, SHANTOU, and SHANWEI, while the clinical symptoms were more severe in the HEYUAN region (Figure 2(b)). These results suggest that the GDHYZ30 strain should be taken seriously in south China since this *C. haemolyticum* could be an important agent of diarrhea and death in piglets and a potential infection risk factor for human beings health in the pork supply as well.

### 3.3. Genome Annotation

**Figure 3:**
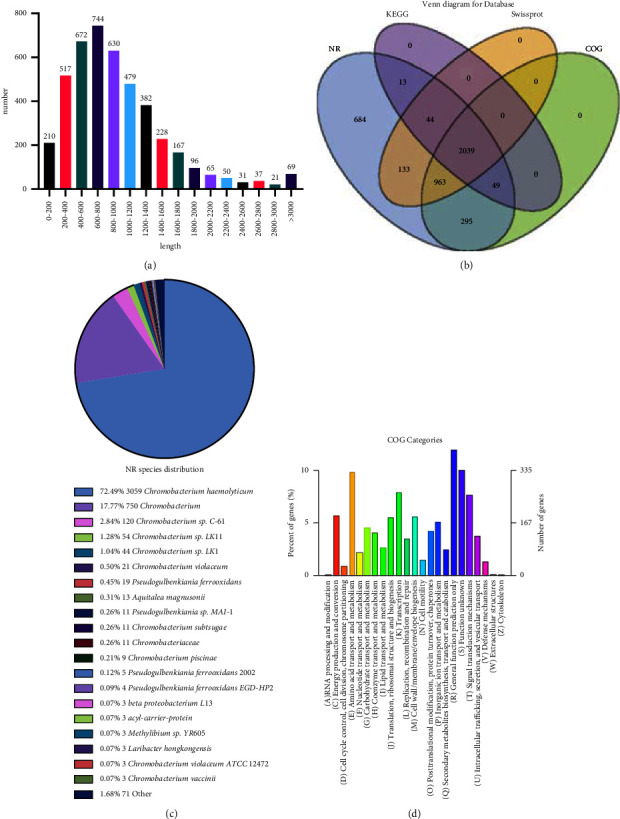
Whole gene annotation. (a) Distribution of gene lengths encoding proteins; (b) Venn diagram of annotated gene function among KEGG, KOG, NR, and Swiss-prot; (c) comparison of homologous genes in NR databases; and (d) COG functional classification of the GDHYZ30.

Whole genome sequencing showed that the genome size of strain GDHYZ30 is 4,785,117 bp with 4,398 coded genes. The genome's GC content is 62.67%, and the longest protein-coding gene is 11,694 bp. The average length of genes that encode a protein is 957 bp, and the proportion of genes encoding proteins was 88.04% (Figure 3(a) and Table 1). After comparing the reference sequences using BLAST x against the NR (NCBI nonredundant protein sequences), Swiss-Prot, COG (Clusters of Orthologous Groups of proteins), and KEGG (Kyoto Encyclopedia of Genes and Genomes) databases. We identified 4,220 unigenes that provided significant results in NR and annotated 3,179, 3,346, and 2,145 unigenes from the Swiss-Prot, COG, and KEGG databases, respectively (Figure 3(b)). By aligning with the NR library, it is possible to view the approximate transcript sequence of the species and the similar species, as well as the functional information of the homologous sequence. The GDHYZ30 strain has 3,059 sequences aligned with the *C. haemolyticum* strain (Figure 3(c)), the most homology among all species analyzed. Functional prediction and classification of unigenes were performed by comparing sequence data against the COG database. A total of 1,935 unigenes were annotated and grouped into 14 categories according to COG function classifications. Among them, the top 3 clusters for general function prediction were “amino acid transport and metabolism” (330 genes), “transcription” (262 genes), and “energy production and conversion” (191 genes) (Figure 3(d)). This whole-genome sequencing project has been deposited at GenBank under the accession number: PRJNA802706.

### 3.4. Antibiotic Susceptibility Assay

Antibiotic susceptibility assays show that the five *C*. *haemolyticum* strains are sensitive to ofloxacin, ciprofloxacin, gentamicin, enrofloxacin, spectinomycin amikacin, and in contrast, they are highly resistant to vancomycin, cefradine, sulfamethoxazole, ampicillin sodium, and sulbactam sodium (Table 2).

### 3.5. Virulence Genes and Drug Resistance

The analysis of drug resistant genes found that the GDHYZ30 strain had 57 drug resistant genes, granting it resistant to *β*-lactams, carbapenem hydrolyzing *β*-lactamases, etc (Supplemental file 2). GDHYZ30 was compared with the SETA and SETB databases, respectively, matching with 390 virulence factors in the SETA database and 432 virulence factors in the SETA database (Supplemental file 3). These results provide a reference for the treatment and pathogenesis of the disease.

### 3.6. Pathogenicity of the GDHYZ30

**Figure 4:**
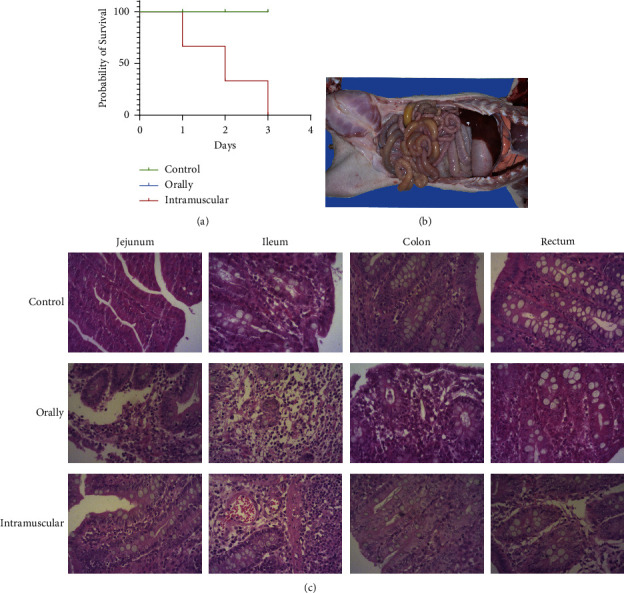
Histopathological changes in experimentally infected with GDHYZ30 strain. (a) Survival rates of piglets in different infected groups; (b) intestinal lesions in piglets with diarrhea; and (c) HE staining were evaluated in intestinal of piglet.

To determine the pathogenicity of the GDHYZ30, Koch postulates experiments were conducted in terms of the GDHYZ30. Nine 30-day-old healthy piglets were randomly divided into three groups. 2 mL of 3.5 × 10^9 CFU bacteria were administered orally or intramuscularly injected, respectively, and 2 mL of PBS was administered orally to the control group. At the second day, we found that all infected groups developed clinical symptoms similar to that of the field cases (Figure 4(b)). One piglet in the intramuscular group had diarrhea and two piglets had died (Figure 4(a)). On the other hand, piglets in the control group were asymptomatic. Thin intestinal walls and flatulence had been observed in piglets with diarrhea from infection groups (Figure 4(b)). In HE staining of intestinal tissues from various infectious groups, exfoliation of epithelial cells and infiltration of inflammatory cells, accompanied by severe ileal bleeding, can be observed. The colon in the irrigation group had more damage, such as inflammatory cell infiltration, than the colon in the intramuscular group (Figure 4(c)). After examination, the GDHYZ30 strain was isolated from the blood and heart samples from the dead piglets, but the GDHYZ30 strain only was isolated from intestinal samples from the oral group. The above results indicated that the GDHYZ30 strain was the pathogen that caused piglet diarrhea and acute death.

## 4. Discussion

Our investigation focused on the frequent outbreaks of diarrhea and death in piglets which drink the mountain spring water, and we isolated a novel hemolytic Gram-negative bacterium in the fecal and drinking water samples of infected piglets, naming it GDHYZ30. 16S rRNA analysis demonstrates that the bacteria were closely related to *C. haemolyticum*. Then we conducted the Koch postulate experiments to confirm that the GDHYZ30 strain was the causative agent of this kind of piglet diarrhea and death. Then the whole gene sequencing analysis of the GDHYZ30 strain was carried out. The phylogenetic tree based on 16S rRNA showed that the GDHYZ30 strain belongs to the same branch as *C. haemolyticum*. At the same time, we isolated *Chromobacterium* in other pig farms where unexplained diarrhea was present, and the homology of these *Chromobacterium* were minor different compared with GDHYZ30 strain. These *Chromobacterium* mainly cause diseases characterized by acute diarrhea and death. It has been reported that *C. haemolyticum* has antibacterial properties, and we speculate that infection with *C. haemolyticum,* except for the direct intestinal damage, may cause changes in the intestinal flora, reducing beneficial bacteria and leading to diarrhea consequently as well. [13]. At the same time, we found that infection with this *C. haemolyticum* can cause death, and the intramuscular injection of the GDHYZ30 strain can even cause acute death in piglets. Meanwhile, the GDHYZ30 strain could be isolated from the blood samples and hearts of dead pigs, while the gavage group only caused diarrhea. We also got similar results when mice were infected with *C. haemolyticum* by irrigation or intramuscular. These results suggest that *C. haemolyticum* can cause bacteremia leading to acute death. The main causative factor may be the hemolytic properties of *C. haemolyticum*. Challenge assays have shown that *C. haemolyticum* can infect piglets through water sources. Reports of human infection with *C. haemolyticum* have been linked to wound exposure to water sources; whether piglets can be infected *C. haemolyticum* through wounds remains to be confirmed [7, 14]. *C. haemolyticum* has different clinical manifestations compared with diarrhea-related pathogens: *Escherichia coli* mainly infects preweaning piglets, while *C. haemolyticum* mainly infects postweaning piglets, and *C. haemolyticum* infection does not cause dehydration in piglets compared with PEDV. The bacteria have 390 virulence genes and 57 drug resistance genes; whether these genes are pathogen-associated still needs further research. Clinical trials and drug resistance analysis show that *C. haemolyticum* is a potential threat to humans and animals. The antibiotic susceptibility assay shows the five isolated *C. haemolyticum* are resistant to most antibiotics, and their susceptibility to some antibiotics varies greatly. Therefore, the drug resistance of the isolates should be measured so that the effective drugs could be administrated.

The growth and development of *C. haemolyticum* require moist water sources. Consequently, the geographical location and climate of Guangdong Province provide an ideal habitat for the reproduction of *C. haemolyticum* [6, 15]. We also detected *C. haemolyticum* in the drinking water of other pig farms with unexplained diarrhea, and after changing the water source, the piglet diarrhea was controlled. Therefore, checking the sanitation of water sources can prevent the occurrence of the disease.

## Figures and Tables

**Table 1:** Genomic sequencing information.

Classes	Names				
	GDHYZ30	GDMMLY	GDHYZ20	GDLF	GDMM22
Size (base)	4785117	5007878	5343702	5158674	4988125
G + C content (%)	62.67	62.39	62.5	62.18	62.34
Protein coding genes	4398	4657	5014	4749	4601
Min length (base)	28	28	28	28	28
Max length (base)	11694	13002	11742	13050	12402
Average length (base)	957.88	947.15	933.19	953.74	954.21
Total coding gene (base)	4212775	4410872	4678995	4529299	4390317
Coding ratio (%)	88.03912	88.07866	87.56093	87.79967	88.01538
tRNA	75	81	79	81	80
rRNA	5	5	4	6	6
Repeat region count	858	0	1084	895	—
Total repeat region (base)	78426	0	72457	56866	0
Repeat ratio (%)	1.64%	0	1.36%	1.10%	0
Low_complexity	21	—	21	19	0
Simple_repeat	793	—	997	858	—
Unknown	44	—	66	18	—

**Table 2:** Antimicrobial sensitivity testing of *C. haemolyticum* (R: resistant).

Drugs	Names				
	GDHYZ30	GDHYZ20	GDMMLY	GDMM22	GDLF
Cefuroxime sodium	R	R	21 mm	13 mm	R
Clindamycin	R	R	R	12 mm	12 mm
Cephalothin	R	R	R	R	R
Cephalexin	R	R	R	R	R
Vancomycin	R	R	R	R	R
Ciprofloxacin	26 mm	30 mm	R	40 mm	41 mm
Ceftriaxone	23 mm	24 mm	21 mm	R	19 mm
Ampicillin sodium and sulbactam sodium	R	R	R	R	R
Spectinomycin	30 mm	21 mm	20 mm	25 mm	24 mm
Ampicillin	R	R	R	R	R
Cefoperazone	19 mm	20 mm	22 mm	R	R
Enrofloxacin	27 mm	25 mm	15 mm	20 mm	32 mm
Novobiocin	27 mm	23 mm	8 mm	22 mm	25 mm
Enoxacin	27 mm	27 mm	9 mm	25 mm	23 mm
Streptomycin	14 mm	17 mm	7 mm	12 mm	16 mm
Bacitracin	16 mm	13 mm	R	R	R
Trimethoprim	R	R	R	23 mm	20 mm
Cefradine	R	R	R	R	R
Penicillin	R	R	R	R	R
Ofloxacin	30 mm	22 mm	23 mm	39 mm	36 mm
Paediatric compound sulfamethoxazole tablets	R	R	R	10 mm	26 mm
Amoxicillin	R	R	R	R	R
Amikacin	22 mm	21 mm	26 mm	20 mm	19 mm
Gentamicin	21 mm	19 mm	17 mm	19 mm	17 mm
Erythromycin	14 mm	12 mm	10 mm	17 mm	18 mm
Florfenicol	19 mm	11 mm	15 mm	21 mm	23 mm
Neomycin	13 mm	14 mm	R	16 mm	16 mm

## Data Availability

This whole-genome sequencing project has been deposited at GenBank under the accession number: PRJNA802706.

## Supplementary Materials

Supplementary Materials The sequence of primers used in this article is listed in Supplemental file.1. We tested the GDHYZ30 strain for drug resistance genes, and the results are listed in Supplemental file.2. The GDHYZ30 stration virulence factor was compared with SETA and SETB databases, the results are listed in Supplemental file.3. Supplemental file.1: Pathogen detection primer sequences. Supplemental file.2: Drug resistance gene statistics. Supplemental file.3: Virulence factor statistics.
