# Supplementary material for: Outbreak of Fatal Piglet Diarrhea Caused by Chromobacterium haemolyticum in China
Source: Transbound Emerg Dis. 2023 Feb 27;2023:6694913. doi: 10.1155/2023/6694913 (PMC12016988; doi:10.1155/2023/6694913)
Supplement: Supplementary Materials — The sequence of primers used in this article is listed in Supplemental file.1. We tested the GDHYZ30 strain for drug resistance genes, and the results are listed in Supplemental file.2. The GDHYZ30 stration virulence factor was compared with SETA and SETB databases, the results are listed in Supplemental file.3. Supplemental file.1: Pathogen detection primer sequences. Supplemental file.2: Drug resistance gene statistics. Supplemental file.3: Virulence factor statistics. [file 6694913.f1.zip › Supplemental file.1 (1).docx]

Supplemental file.1: Primers used for detecting diarrhea-associated viruses and main virus.

| Name | Sequence(5′-3′) |
| --- | --- |
| CSFV-F | CAGGTATGCGATCTCGTCAACCA |
| CSFV-R | GGGCACAGCCCAAATCCGAAGT |
| PBoV-F | ACAGGCAGCCGATCACTCACTAT |
| PBoV-R | CTCGTTCCTCCCATCAGACACTT |
| PCLV-F | TTAACACGGCGAGTATATCTACCAGA |
| PCLV-R | GTCTCAGGACTTCGATGTATTCGACCCTT |
| PCV2-F | CTCTGAATTGTACATACATGGTTACACGGA |
| PCV2-R | TGACGTATCCAAGGAGGCGTTA |
| PCV3-F | CCACAGAAGGCGCTATGTC |
| PCV3-R | CCGCATAAGGGTCGTCTTG |
| PCV4-F | GCAGTAATGACGTAGTCCCGGAG |
| PCV4-R | CAGCGACCTTAAAGCGGCTGTG |
| PDCoV-F | TTTCAGGTGCTCAAAGCTCA |
| PDCoV-R | GTTAACAGATTGAGATCTTGG |
| PEDV-F | GTCTTACTGCGAATTGACC |
| PEDV-R | GGCATAGAGAGATAATGGCA |
| PKOV-F | GGCATTGACATGAATCAGGC |
| PKOV-R | GCGATCGTAGGTCTTCGG |
| PRoV-F | GTATGGTATTGAATATACC |
| PRoV-R | TAGACTGATCCAGTTGGC |
| PRRSV-F | CATTATTGGCGTGTAGGTGATAGAAAA |
| PRRSV-F | GTGTCAGGCATTGTGGCTGTGT |
| PRV-F | TCCACTCGCAGCTCTTCT |
| PRV-R | GCACGTCATCACGAAGGA |
| PSV-F | GATGTGGCGCATGCTCTT |
| PSV-R | TGCTGCCTCCTGTGTTGTTAT |
| PTV-F | TGAAAGACCTGCTCTGGCGCGAG |
| PTV-R | GCTGGTGGGCCCCAGAGAAATCTC |
| SADS-CoV-R | GTGCTACGTAAGTAGTGTCGT |
| SADS-CoV-F | ATGAAACTTTTTACAGTTTTCACGC |
| TGEV-F | GATGGCGACCAGATAGAAGT |
| TGEV-R | GCAATAGGGTTGCTTGTACC |
| 16S-F | AGAGTTTGATCCTCCTGGCTTAG |
| 16S-R | TGACGGGCGGTGTGTACAA |

Note:CSFV, classical swine fever virus; PBoV, porcine bocavirus; PCLV, porcine circovirus-like viruses; PCV2, porcine circovirus type 2; PCV3, porcine circovirus type 3; PCV4, porcine circovirus type 4; PDCoV, porcine deltacoronavirus; PEDV, porcine epidemic diarrhoea virus; PKOV, porcine Kobuviruses; PRoV, porcine rotavirus; PRRSV, porcine reproductive and respiratory syndrome virus; PRV, Pseudorabies virus; PSV, porcine sapelovirus; PTV, porcine teschovirus; SADS-CoV, swine acute diarrhoea syndrome coronavirus; TGEV, porcine transmissible gastroenteritis virus.
