# Supplementary material for: Outbreak of Fatal Piglet Diarrhea Caused by Chromobacterium haemolyticum in China
Source: Transbound Emerg Dis. 2023 Feb 27;2023:6694913. doi: 10.1155/2023/6694913 (PMC12016988; doi:10.1155/2023/6694913)
Supplement: Supplementary Materials — The sequence of primers used in this article is listed in Supplemental file.1. We tested the GDHYZ30 strain for drug resistance genes, and the results are listed in Supplemental file.2. The GDHYZ30 stration virulence factor was compared with SETA and SETB databases, the results are listed in Supplemental file.3. Supplemental file.1: Pathogen detection primer sequences. Supplemental file.2: Drug resistance gene statistics. Supplemental file.3: Virulence factor statistics. [file 6694913.f1.zip › Supplemental file.2.pdf]

Supplemental file.2: Drug resistance gene statistics

Prot	Info	ARO_Nam	ARO_count
PROKKA_	Multidrug resistance protein Md	acrA;acrA;	2
PROKKA_	Undecaprenyl-phosphate 4-deox	PmrF;	1
PROKKA_	Aminoglycoside N(6')-acetyltrar	AAC(6')-Iy	15
PROKKA_	Efflux pump membrane transpoi	cmeB;ceoI	20
PROKKA_	Bifunctional polymyxin resistan	arnA;arnA	2
PROKKA_	UDP-N-acetylglucosamine 1-cai	Escherichi	2
PROKKA_	DNA gyrase subunit A	Pseudomoi	6
PROKKA_	Multidrug transporter MdfA	mdfA;	1
PROKKA_	Transcriptional regulatory prote	mtrA;	1
PROKKA_	Cobalt-zinc-cadmium resistance	mexK;TriC	2
PROKKA_	Beta-lactamase	y56 beta-la	328
PROKKA_	CDP-diacylglycerol--glycerol-3-	Staphylocc	1
PROKKA_	Swarming motility regulation pr	PmrA;	1
PROKKA_	Carbepenem-hydrolyzing beta-la	KPC-5;KP	120
PROKKA_	Transcription-repair-coupling fa	mfd;	1
PROKKA_	Efflux pump periplasmic linker	acrA;	1
PROKKA_	Transcriptional regulatory prote	PmrA;Pseu	2
PROKKA_	Transcriptional regulatory prote	cpxR;	1
PROKKA_	Transcriptional regulatory prote	PmrA;Pseu	4
PROKKA_	Fosmidomycin resistance protei	rosA;	1
PROKKA_	Dihydropteroate synthase	Escherichi	3
PROKKA_	Macrolide export ATP-binding/	macB;	1
PROKKA_	Elongation factor Tu	Escherichi	7
PROKKA_	Undecaprenyl-diphosphatase	bacA;	1
PROKKA_	DNA-directed RNA polymerase	Escherichi	1
PROKKA_	DNA topoisomerase 4 subunit E	Salmonella	3
PROKKA_	Transcriptional regulatory prote	PmrA;	1
PROKKA_	Multidrug resistance protein Md	mdtC;mdtI	3
PROKKA_	UDP-3-O-[3-hydroxymyristoyl]	LpxC;	1
PROKKA_	Dihydrofolate reductase	dfrA3;dfrA	6
PROKKA_	Glutathione transferase FosA	FosA;FosA	7
PROKKA_	Efflux pump membrane transpoi	cmeB;acrD	20
PROKKA_	Beta-lactamase	SRT-2;SRT	27
PROKKA_	HTH-type transcriptional regula	aminocoun	1
PROKKA_	HTH-type transcriptional regula	mexZ;	1
PROKKA_	KDP operon transcriptional regu	kdpE;	1
PROKKA_	Transcriptional regulatory prote	smeR;baeF	2
PROKKA_	Multidrug transporter EmrE	qacH;abeS	4
PROKKA_	Multidrug resistance protein Md	mexN;mdt	3
PROKKA_	Acyl-[acyl-carrier-protein]--UDI	LpxA;	1
PROKKA_	Multidrug export protein EmrB	emrB;emrY	2
PROKKA_	DNA gyrase subunit B	Escherichi	4
PROKKA_	Multidrug export protein EmrA	emrA;	1
PROKKA_	30S ribosomal protein S12	Mycobacte	1
PROKKA_	Glycerol-3-phosphate transporte	Escherichi	1
PROKKA_	Phosphate regulon transcription	arlR;vanRI	6
PROKKA_	Multidrug resistance protein Md	mdtB;mdtC	3
PROKKA_	putative ABC transporter ATP-b	macB;	1
PROKKA_	Alanine--tRNA ligase	aminocoun	1
PROKKA_	Transcriptional repressor MprA	emrR;	1
PROKKA_	Elongation factor Tu	Escherichi	7
PROKKA_	DNA topoisomerase 4 subunit A	Escherichi	2
PROKKA_	Catalase-peroxidase	Mycobacte	1
PROKKA_	Transcriptional regulatory prote	vanRI;	1
PROKKA_	Multidrug resistance protein Md	mdtH;	1
PROKKA_	16S/23S rRNA (cytidine-2'-O)-r	Mycobacte	1

PROKKA_	Beta-lactamase OXA-1 precursc	OXA-59;C	9
