# Supplementary material for: Outbreak of Fatal Piglet Diarrhea Caused by Chromobacterium haemolyticum in China
Source: Transbound Emerg Dis. 2023 Feb 27;2023:6694913. doi: 10.1155/2023/6694913 (PMC12016988; doi:10.1155/2023/6694913)
Supplement: Supplementary Materials — The sequence of primers used in this article is listed in Supplemental file.1. We tested the GDHYZ30 strain for drug resistance genes, and the results are listed in Supplemental file.2. The GDHYZ30 stration virulence factor was compared with SETA and SETB databases, the results are listed in Supplemental file.3. Supplemental file.1: Pathogen detection primer sequences. Supplemental file.2: Drug resistance gene statistics. Supplemental file.3: Virulence factor statistics. [file 6694913.f1.zip › Supplemental file.3.pdf]

Supplemental file.3: Virulence factor statistics

setA

Prot	Info	VF_terms	VF_Names	VF_count
PROKKA_	Isochorism	VF0228;	Enterobact	1
PROKKA_	Flagellar b	VF0157;V	Flagella;Fl	4
PROKKA_	Spermidin	VF0268;	HitABC;	1
PROKKA_	Flagellar h	VF0394;	Flagella;	1
PROKKA_	RNA polyı	VF0430;V	Flagella;Fl	2
PROKKA_	Transcripti	VF0082;	Type IV pi	1
PROKKA_	Urease sub	VF0050;	Urease;	1
PROKKA_	putative ox	VF0033;	LPS;	1
PROKKA_	3-oxoacyl-	VF0228;	Enterobact	1
PROKKA_	Chemotaxi	VF0430;	Flagella;	1
PROKKA_	Heme/hem	VF0269;V	HxuABC;I	2
PROKKA_	UDP-gluco	VF0003;	Capsule;	1
PROKKA_	Putative 2-	VF0272;	FbpABC;	1
PROKKA_	Type II sec	VF0084;V	xcp secreti	3
PROKKA_	Glutamine	VF0272;V	FbpABC;Y	6
PROKKA_	Taurine im	VF0095;	Pyochelin;	1
PROKKA_	Invasion pı	VF0116;V	TTSS(SPI-	15
PROKKA_	Iron(3+)-h	VF0095;	Pyochelin;	1
PROKKA_	Multidrug	VF0451;	MtrCDE;	1
PROKKA_	Transcripti	VF0286;V	PhoP;Flage	2
PROKKA_	UTP--gluc	VF0244;	Hyaluronic	1
PROKKA_	Lipoprotei	VF0272;V	FbpABC;F	14
PROKKA_	Flagellar b	VF0273;V	Flagella;Fl	10
PROKKA_	Phosphohe	VF0436;V	Capsule I;(	2
PROKKA_	Chemotaxi	VF0430;	Flagella;	1
PROKKA_	Multidrug	VF0451;	MtrCDE;	1
PROKKA_	Salmonella	VF0116;	TTSS(SPI-	1
PROKKA_	Glucose-1-	VF0436;	Capsule I;	1
PROKKA_	Methyl-acc	VF0127;	ACF;	1
PROKKA_	Flagellar L	VF0273;V	Flagella;Fl	4
PROKKA_	ATP-deper	VF0072;	ClpC;	1
PROKKA_	Spermidin	VF0268;	HitABC;	1
PROKKA_	Flagellar h	VF0273;V	Flagella;Fl	2
PROKKA_	ABC trans	VF0256;V	Shu;Chu;Y	4
PROKKA_	Twitching	VF0082;	Type IV pi	1
PROKKA_	UDP-2-ace	VF0033;V	LPS;LPS;I	3
PROKKA_	Major exp	VF0334;	HSI-I;	1
PROKKA_	Dimodular	VF0228;	Enterobact	1
PROKKA_	Macrolide	VF0451;	MtrCDE;	1
PROKKA_	Oxygen-re	VF0116;V	TTSS(SPI-	2
PROKKA_	Flagellar M	VF0430;V	Flagella;Fl	5
PROKKA_	Flagellar b	VF0273;V	Flagella;Fl	5
PROKKA_	Outer mem	VF0105;V	Lpf;Haema	14
PROKKA_	Chemotaxi	VF0430;	Flagella;	1
PROKKA_	D-inositol-	VF0367;	LPS;	1
PROKKA_	Flagellar F	VF0157;	Flagella;	1
PROKKA_	Flagellar L	VF0394;V	Flagella;Fl	4
PROKKA_	Transcripti	VF0091;	Alginate;	1
PROKKA_	3-oxoacyl-	VF0436;V	Capsule I;Y	2
PROKKA_	Major oute	VF0081;	Porin;	1
PROKKA_	Response ı	VF0428;	Bsa T3SS;	1
PROKKA_	hypothetica	VF0334;	HSI-I;	1
PROKKA_	Competenc	VF0082;	Type IV pi	1
PROKKA_	Levodione	VF0228;	Enterobact	1
PROKKA_	putative m	VF0136;V	Yersiniaba	2

PROKKA_	Iron impor	VF0136;	Yersiniaba	1
PROKKA_	Response r	VF0428;V	Bsa T3SS;	2
PROKKA_	flagellar bi	VF0273;V	Flagella;Fl	5
PROKKA_	Motility pr	VF0157;V	Flagella;Fl	2
PROKKA_	4-carboxy-	VF0033;	LPS;	1
PROKKA_	flagellar ba	VF0273;V	Flagella;Fl	2
PROKKA_	Transcripti	VF0428;	Bsa T3SS;	1
PROKKA_	Putative ph	VF0268;V	HitABC;Fl	5
PROKKA_	Motility pr	VF0430;	Flagella;	1
PROKKA_	UDP-N-ac	VF0003;	Capsule;	1
PROKKA_	RNA polyi	VF0394;V	Flagella;Fl	3
PROKKA_	Inositol ph	VF0116;	TTSS(SPI-	1
PROKKA_	High-affini	VF0268;V	HitABC;Fl	9
PROKKA_	Transcripti	VF0430;	Flagella;	1
PROKKA_	Peptide me	VF0456;	MsrAB;	1
PROKKA_	Cell invasi	VF0116;	TTSS(SPI-	1
PROKKA_	Flagellar b	VF0116;V	TTSS(SPI-	13
PROKKA_	Flagellar m	VF0430;V	Flagella;Fl	5
PROKKA_	Flagellar h	VF0273;V	Flagella;Fl	3
PROKKA_	hypothetica	VF0334;V	HSI-I;T6SS	2
PROKKA_	Enoyl-[acy	VF0228;	Enterobact	1
PROKKA_	Basal-body	VF0430;V	Flagella;Fl	2
PROKKA_	Major oute	VF0081;	Porin;	1
PROKKA_	Flagellar m	VF0273;	Flagella;	1
PROKKA_	ATP synth	VF0035;	TTSS;	1
PROKKA_	C4-dicarbo	VF0082;V	Type IV pi	4
PROKKA_	putative ox	VF0228;V	Enterobact	2
PROKKA_	Laminin-bi	VF0221;V	Type 1 fim	14
PROKKA_	Cholix tox	VF0086;	ExoA;	1
PROKKA_	Chaperone	VF0334;V	HSI-I;T6SS	2
PROKKA_	Putative m	VF0225;V	Hemolysin	4
PROKKA_	Phenyloxa	VF0094;	Pyoverdine	1
PROKKA_	ABC trans	VF0272;V	FbpABC;C	3
PROKKA_	Chaperone	VF0334;V	HSI-I;T6SS	3
PROKKA_	Flagellar b	VF0430;V	Flagella;Fl	6
PROKKA_	Flagellar b	VF0273;V	Flagella;Fl	7
PROKKA_	CDP-gluco	VF0392;V	O-antigen;	2
PROKKA_	2-hydroxyı	VF0089;	Rhamnolip	1
PROKKA_	L-ornithine	VF0094;V	Pyoverdine	3
PROKKA_	D-methion	VF0513;	IlpA;	1
PROKKA_	Phosphogl	VF0392;	O-antigen;	1
PROKKA_	Superoxide	VF0169;	SodB;	1
PROKKA_	Flagellar b	VF0430;V	Flagella;Fl	5
PROKKA_	UDP-N-ac	VF0033;	LPS;	1
PROKKA_	ADP-L-gly	VF0044;V	LOS;LOS;	3
PROKKA_	hypothetica	VF0334;V	HSI-I;T6SS	3
PROKKA_	Chemotaxi	VF0157;V	Flagella;Fl	3
PROKKA_	Flagellar h	VF0273;V	Flagella;Fl	3
PROKKA_	Flagellar b	VF0430;V	Flagella;Fl	9
PROKKA_	Hemin trar	VF0227;V	Chu;Shu;	2
PROKKA_	Undecapre	VF0124;	LPS;	1
PROKKA_	Efflux pun	VF0451;	MtrCDE;	1
PROKKA_	Intracellula	VF0334;	HSI-I;	1
PROKKA_	Putative m	VF0430;	Flagella;	1
PROKKA_	Sulfate/thi	VF0268;	HitABC;	1
PROKKA_	Arabinose	VF0095;	Pyochelin;	1
PROKKA_	Transcripti	VF0428;	Bsa T3SS;	1
PROKKA_	3-oxoacyl-	VF0228;	Enterobact	1

PROKKA_	Alpha-D-ri	VF0272;V	FbpABC;Y	2
PROKKA_	N,N'-diace	VF0079;V	Capsule;L(	2
PROKKA_	Transcripti	VF0091;	Alginate;	1
PROKKA_	Phosphate	VF0228;V	Enterobact	12
PROKKA_	Flagellar b	VF0114;	Flagella;	1
PROKKA_	hypothetic	VF0334;V	HSI-I;T6S	2
PROKKA_	putative A	VF0136;	Yersiniaba	1
PROKKA_	Transcripti	VF0286;	PhoP;	1
PROKKA_	Carnitine c	VF0414;	RicA;	1
PROKKA_	Flagellin B	VF0394;	Flagella;	1
PROKKA_	Malonyl C	VF0136;V	Yersiniaba	2
PROKKA_	GDP-manr	VF0392;V	O-antigen;	5
PROKKA_	60 kDa ch	VF0159;	Hsp60;	1
PROKKA_	Chaperone	VF0224;V	F1C fimbr	7
PROKKA_	Chemotaxi	VF0430;V	Flagella;Pl	3
PROKKA_	Sensory tr	VF0286;	PhoP;	1
PROKKA_	hypothetic	VF0429;	T6SS-1;	1
PROKKA_	Methyl-ac	VF0430;	Flagella;	1
PROKKA_	Flagellum-	VF0273;V	Flagella;Fl	14
PROKKA_	Colicin I r	VF0228;V	Enterobact	3
PROKKA_	Multidrug	VF0450;	FarAB;	1
PROKKA_	Lipopolys	VF0056;V	LPS;LOS;	2
PROKKA_	Salicylate	VF0095;	Pyochelin;	1
PROKKA_	chemotaxi	VF0430;	Flagella;	1
PROKKA_	Oxygen se	VF0432;	CdpA;	1
PROKKA_	Chaperone	VF0116;	TTSS(SPI-	1
PROKKA_	Motility p	VF0430;V	Flagella;Fl	2
PROKKA_	O-GlcNAc	VF0389;	mu-toxin;	1
PROKKA_	Yqey-like	VF0156;	Dot/Icm;	1
PROKKA_	Chaperone	VF0072;V	ClpC;HSI-	3
PROKKA_	Arabinose	VF0082;	Type IV pi	1
PROKKA_	DNA repai	VF0457;	RecN;	1
PROKKA_	Ditrans,po	VF0361;	Capsule;	1
PROKKA_	Multidrug	VF0451;	MtrCDE;	1
PROKKA_	Sigma fact	VF0091;	Alginate;	1
PROKKA_	Iron impor	VF0136;V	Yersiniaba	2
PROKKA_	Response r	VF0082;	Type IV pi	1
PROKKA_	Chemotaxi	VF0430;	Flagella;	1
PROKKA_	Transcripti	VF0286;	PhoP;	1
PROKKA_	Sulfate tra	VF0272;	FbpABC;	1
PROKKA_	hypothetic	VF0429;V	T6SS-1;T6	2
PROKKA_	Type-1 fin	VF0031;V	Fimbriae;F	13
PROKKA_	Twitching	VF0082;V	Type IV pi	3
PROKKA_	Type II sec	VF0084;V	xcp secreti	7
PROKKA_	Linear gra	VF0299;	Mycobacti	1
PROKKA_	hypothetic	VF0430;V	Flagella;Fl	7
PROKKA_	Hemin imp	VF0227;V	Chu;Shu;E	4
PROKKA_	Flagellar h	VF0430;V	Flagella;Fl	4
PROKKA_	Swarming	VF0286;	PhoP;	1
PROKKA_	Flagellar P	VF0273;V	Flagella;Fl	5
PROKKA_	D-beta-D-l	VF0044;V	LOS;LOS;	2
PROKKA_	2,3-dihydr	VF0095;V	Pyochelin;	7
PROKKA_	8-amino-7-	VF0436;	Capsule I;	1
PROKKA_	Flagellar P	VF0430;V	Flagella;Fl	5
PROKKA_	putative A	VF0225;V	Hemolysin	2
PROKKA_	Methyl-ac	VF0430;	Flagella;	1
PROKKA_	Alginate b	VF0436;V	Capsule I;A	5
PROKKA_	Yop protei	VF0116;	TTSS(SPI-	1

PROKKA_	Type II sec	VF0082;V	Type IV pi	3
PROKKA_	Catalase pı	VF0454;	KatA;	1
PROKKA_	N-acylneu	VF0274;V	Capsule;Ca	4
PROKKA_	Alpha-D-k	VF0033;V	LPS;Capsu	2
PROKKA_	Chaperone	VF0036;V	Haemagglu	7
PROKKA_	hypothetic	VF0334;	HSI-I;	1
PROKKA_	Flagellar b	VF0430;V	Flagella;Fl	15
PROKKA_	Transcripti	VF0091;	Alginate;	1
PROKKA_	Flagellar h	VF0273;V	Flagella;Fl	4
PROKKA_	Flagellum-	VF0430;V	Flagella;Fl	12
PROKKA_	hypothetic	VF0334;	HSI-I;	1
PROKKA_	Lipid A ex	VF0225;V	Hemolysin	4
PROKKA_	Biofilm op	VF0014;	Intercellula	1
PROKKA_	colanic aci	VF0003;	Capsule;	1
PROKKA_	Transcripti	VF0273;V	Flagella;Ty	3
PROKKA_	Transcripti	VF0286;	PhoP;	1
PROKKA_	Fimbrial as	VF0082;	Type IV pi	1
PROKKA_	Di-/tripept	VF0293;	IraAB;	1
PROKKA_	D-beta-hyd	VF0228;V	Enterobact	2
PROKKA_	Putative ty	VF0154;	<i>lsp</i>	1
PROKKA_	hypothetic	VF0156;	Dot/Icm;	1
PROKKA_	hypothetic	VF0334;V	HSI-I;T6S	2
PROKKA_	TonB-depe	VF0269;V	HxuABC;C	3
PROKKA_	putative si	VF0256;V	Shu;Chu;	2
PROKKA_	L-lysine N	VF0229;V	Aerobactin	3
PROKKA_	Phosphate	VF0286;	PhoP;	1
PROKKA_	Lipoprotei	VF0272;V	FbpABC;H	6
PROKKA_	hypothetic	VF0429;V	T6SS-1;T6	2
PROKKA_	putative A	VF0268;V	HitABC;Fl	10
PROKKA_	UDP-N-ac	VF0274;	Capsule;	1
PROKKA_	Type III se	VF0118;V	TTSS;Bsa	2
PROKKA_	Chemotaxi	VF0430;	Flagella;	1
PROKKA_	Outer mem	VF0224;V	F1C fimbri	9
PROKKA_	dTDP-gluc	VF0436;V	Capsule I;V	6
PROKKA_	Chaperone	VF0031;V	Fimbriae;T	9
PROKKA_	Cell divisi	VF0268;V	HitABC;C	18
PROKKA_	UDP-4-am	VF0033;V	LPS;LPS;I	3
PROKKA_	High-affini	VF0228;V	Enterobact	8
PROKKA_	Urease sub	VF0050;	Urease;	1
PROKKA_	Hemin-bin	VF0256;V	Shu;Chu;	2
PROKKA_	Outer mem	VF0451;	MtrCDE;	1
PROKKA_	Flagellar h	VF0394;	Flagella;	1
PROKKA_	Transcripti	VF0286;	PhoP;	1
PROKKA_	Cobalt-zin	VF0451;	MtrCDE;	1
PROKKA_	Outer mem	VF0451;	MtrCDE;	1
PROKKA_	Lipoprotei	VF0116;V	TTSS(SPI-	3
PROKKA_	Outer mem	VF0221;V	Type 1 fim	8
PROKKA_	Virulence	VF0286;	PhoP;	1
PROKKA_	Putative ty	VF0154;V	<i>lsp</i>	3
PROKKA_	CDP-parat	VF0436;V	Capsule I;C	8
PROKKA_	Outer mem	VF0220;V	P fimbriae;	15
PROKKA_	Transcripti	VF0286;V	PhoP;Bsa T	2
PROKKA_	ECF RNA	VF0091;	Alginate;	1
PROKKA_	Transcripti	VF0286;V	PhoP;Algin	2
PROKKA_	3-oxoacyl-	VF0228;V	Enterobact	2
PROKKA_	Octopine p	VF0268;V	HitABC;Fl	11
PROKKA_	Putative gl	VF0326;	LOS;	1
PROKKA_	Pseudolysi	VF0087;	LasB;	1

PROKKA_	hypothetic	VF0334;V	HSI-I;T6S	3
PROKKA_	Lipopolysa	VF0268;V	HitABC;Fl	10
PROKKA_	Glucose 1-	VF0228;V	Enterobact	2
PROKKA_	Transcripti	VF0428;	Bsa T3SS;	1
PROKKA_	Vitamin B	VF0256;	Shu;	1
PROKKA_	Spermidin	VF0268;	HitABC;	1
PROKKA_	N,N'-diace	VF0079;V	Capsule;L	2
PROKKA_	Major oute	VF0081;	Porin;	1
PROKKA_	Guanosine	VF0287;	RelA;	1
PROKKA_	hypothetic	VF0334;	HSI-I;	1
PROKKA_	hypothetic	VF0429;V	T6SS-1;T6	2
PROKKA_	Motility pr	VF0430;V	Flagella;Fl	2
PROKKA_	putative pe	VF0455;V	MntABC;I	5
PROKKA_	GDP-manr	VF0367;	LPS;	1
PROKKA_	Outer mem	VF0116;V	TTSS(SPI-	5
PROKKA_	Type II sec	VF0084;V	xcp secreti	3
PROKKA_	Glycerol-3	VF0264;	Hpt;	1
PROKKA_	Multidrug	VF0451;	MtrCDE;	1
PROKKA_	Cell invasi	VF0116;V	TTSS(SPI-	3
PROKKA_	3-oxoacyl-	VF0228;	Enterobact	1
PROKKA_	hypothetic	VF0334;V	HSI-I;T6S	3
PROKKA_	Type IV pi	VF0082;	Type IV pi	1
PROKKA_	Outer mem	VF0451;	MtrCDE;	1
PROKKA_	3-oxoacyl-	VF0228;	Enterobact	1
PROKKA_	p-hydroxyl	VF0450;V	FarAB;Do	2
PROKKA_	NAD-depe	VF0444;	Lap;	1
PROKKA_	3-oxoacyl-	VF0228;	Enterobact	1
PROKKA_	Catalase-p	VF0168;V	KatAB;Ka	2
PROKKA_	Flagellar b	VF0157;V	Flagella;Fl	7
PROKKA_	HTH-type	VF0362;	BopD;	1
PROKKA_	Type 4 pre	VF0082;V	Type IV pi	2
PROKKA_	Glucose 1-	VF0228;	Enterobact	1
PROKKA_	GDP-L-fuc	VF0392;V	O-antigen;	4
PROKKA_	flagellar bi	VF0116;V	TTSS(SPI-	4
PROKKA_	putative bi	VF0272;	FbpABC;	1
PROKKA_	High-affin	VF0272;V	FbpABC;F	10
PROKKA_	UDP-4-am	VF0033;V	LPS;LPS;I	3
PROKKA_	Bifunction	VF0323;V	Capsule;Ca	3
PROKKA_	Multidrug	VF0451;	MtrCDE;	1
PROKKA_	Manganes	VF0455;	MntABC;	1
PROKKA_	hypothetic	VF0334;	HSI-I;	1
PROKKA_	Phthiocero	VF0436;	Capsule I;	1
PROKKA_	UDP-N-ac	VF0091;	Alginate;	1
PROKKA_	putative ox	VF0228;	Enterobact	1
PROKKA_	Peptidogly	VF0430;	Flagella;	1
PROKKA_	Isochorism	VF0228;	Enterobact	1
PROKKA_	Flagellar n	VF0430;V	Flagella;Fl	5
PROKKA_	putative si	VF0361;	Capsule;	1
PROKKA_	hypothetic	VF0334;V	HSI-I;T6S	3
PROKKA_	UDP-2-ace	VF0033;	LPS;	1
PROKKA_	UDP-3-O-[	VF0436;	Capsule I;	1
PROKKA_	Non-hemo	VF0092;	PLC;	1
PROKKA_	dTDP-4-de	VF0392;	O-antigen;	1
PROKKA_	Type II sec	VF0155;V	Type IV pi	7
PROKKA_	Histidinol-	VF0323;V	Capsule;Ca	2
PROKKA_	3-oxoacyl-	VF0228;V	Enterobact	2
PROKKA_	Flagellar b	VF0430;V	Flagella;Fl	9
PROKKA_	Long-chai	VF0095;V	Pyochelin;	7

PROKKA_	Type-1 fim	VF0105;V	Lpf;S fimb	12
PROKKA_	Urease acc	VF0050;	Urease;	1
PROKKA_	Methylisoc	VF0253;	Isocitrate l	1
PROKKA_	Chaperone	VF0031;V	Fimbriae;F	9
PROKKA_	Alginate bi	VF0082;V	Type IV pi	3
PROKKA_	Flagellar b	VF0273;V	Flagella;Fl	14
PROKKA_	Dimodular	VF0228;V	Enterobact	2
PROKKA_	Aldehyde-a	VF0444;	Lap;	1
PROKKA_	Outer mem	VF0105;V	Lpf;F1C fi	15
PROKKA_	Poly-beta-1	VF0014;	Intercellula	1
PROKKA_	Alcohol de	VF0444;	Lap;	1
PROKKA_	Flagellar b	VF0394;V	Flagella;Fl	3
PROKKA_	hypothetica	VF0334;	HSI-I;	1
PROKKA_	Organic hy	VF0222;	S fimbriae;	1
PROKKA_	Twitching	VF0082;	Type IV pi	1
PROKKA_	Nitrogen re	VF0082;	Type IV pi	1
PROKKA_	Flagellar b	VF0273;V	Flagella;Fl	9
PROKKA_	putative A'	VF0116;V	TTSS(SPI-	14
PROKKA_	Chaperone	VF0334;V	HSI-I;T6SS	3
PROKKA_	Flagellar h	VF0394;	Flagella;	1
PROKKA_	60 kDa cha	VF0159;	Hsp60;	1
PROKKA_	Chemotaxi	VF0430;	Flagella;	1
PROKKA_	6-hydroxyn	VF0100;	Pyocyanin;	1
PROKKA_	Type-1 fim	VF0105;V	Lpf;S fimb	8
PROKKA_	putative ef	VF0451;	MtrCDE;	1
PROKKA_	HrpJ-like c	VF0118;	TTSS;	1
PROKKA_	Flagellar b	VF0273;	Flagella;	1
PROKKA_	Multidrug	VF0028;V	Cya;Hemo	3
PROKKA_	Long-chain	VF0095;V	Pyochelin;	6
PROKKA_	Chemotaxi	VF0430;	Flagella;	1
PROKKA_	Sensor pro	VF0082;	Type IV pi	1
PROKKA_	Efflux pun	VF0451;	MtrCDE;	1
PROKKA_	Arabinose	VF0323;	Capsule;	1
PROKKA_	Transcripti	VF0286;	PhoP;	1
PROKKA_	hypothetica	VF0105;	Lpf;	1
PROKKA_	Hemin tran	VF0256;V	Shu;Chu;	2
PROKKA_	Putative be	VF0163;V	enh loci;en	2
PROKKA_	Histidine t	VF0272;V	FbpABC;F	13
PROKKA_	Flagellar b	VF0430;V	Flagella;Fl	5
PROKKA_	Multidrug	VF0451;	MtrCDE;	1
PROKKA_	Lipopolysa	VF0085;V	LPS;LPS;	2
PROKKA_	putative To	VF0094;V	Pyoverdine	2
PROKKA_	Flagellar h	VF0273;V	Flagella;Fl	3
PROKKA_	Long-chain	VF0095;V	Pyochelin;	10
PROKKA_	FK506-bin	VF0153;	Mip;	1
PROKKA_	Sorbitol de	VF0228;V	Enterobact	2
PROKKA_	2-C-methy	VF0043;	Capsule;	1
PROKKA_	Alginate bi	VF0091;V	Alginate;C	5
PROKKA_	Acetoacety	VF0228;V	Enterobact	2
PROKKA_	flagellar ba	VF0157;	Flagella;	1
PROKKA_	Methyl-acc	VF0126;	TCP;	1
PROKKA_	Urease sub	VF0050;	Urease;	1
PROKKA_	Outer mem	VF0221;V	Type 1 fim	14
PROKKA_	Galactose/	VF0268;V	HitABC;Fl	3
PROKKA_	Isocitrate l	VF0253;	Isocitrate l	1
PROKKA_	Transcripti	VF0428;	Bsa T3SS;	1
PROKKA_	Transcripti	VF0082;V	Type IV pi	3
PROKKA_	Phosphohe	VF0326;V	LOS;Capsu	3

PROKKA_	2,3-dihydr	VF0228;V	Enterobact	2
PROKKA_	Surface pr	VF0428;V	Bsa T3SS;	5
PROKKA_	UDP-N-ac	VF0144;V	Capsule;Ca	4
PROKKA_	Outer mem	VF0322;	CadF;	1
PROKKA_	HTH-type	VF0362;	BopD;	1
PROKKA_	3-deoxy-D	VF0085;V	LPS;LOS;	2
PROKKA_	Magnesium	VF0106;	MgtBC;	1
PROKKA_	CDP-abequ	VF0392;	O-antigen;	1
PROKKA_	Ribosomal	VF0156;	Dot/Icm;	1
PROKKA_	Trifunction	VF0422;	AdsA;	1
PROKKA_	Fe(3+) ion	VF0272;	FbpABC;	1
PROKKA_	Methionine	VF0268;V	HitABC;C	9
PROKKA_	Lipopolysa	VF0272;V	FbpABC;F	5
PROKKA_	flagellar bi	VF0116;V	TTSS(SPI-	4
PROKKA_	Lipid A bi	VF0326;	LOS;	1
PROKKA_	Outer mem	VF0222;V	S fimbriae;	15
PROKKA_	Flagellar b	VF0273;V	Flagella;Fl	4
PROKKA_	ECF RNA	VF0091;	Alginate;	1
PROKKA_	Flagellar M	VF0273;V	Flagella;Fl	3
PROKKA_	Endonucle	VF0422;	AdsA;	1
PROKKA_	Pilus assem	VF0082;	Type IV pi	1
PROKKA_	putative m	VF0082;	Type IV pi	1
PROKKA_	Linear gra	VF0136;	Yersiniaba	1
PROKKA_	KDP operc	VF0286;	PhoP;	1
PROKKA_	Hemin tran	VF0256;V	Shu;Chu;E	4
PROKKA_	3-oxoacyl-	VF0436;	Capsule I;	1
PROKKA_	ADP-hepto	VF0085;V	LPS;LOS;I	3
PROKKA_	GTP pyrop	VF0287;	RelA;	1
PROKKA_	Chemotaxi	VF0430;	Flagella;	1
PROKKA_	Dimodular	VF0094;	Pyoverdine	1
PROKKA_	Colicin I re	VF0048;V	HmbR;Hpu	5
PROKKA_	Type II sec	VF0084;	xcp secreti	1
PROKKA_	Lipopolysa	VF0085;	LPS;	1
PROKKA_	Type III se	VF0116;V	TTSS(SPI-	2
PROKKA_	Ferrous iro	VF0160;	FeoAB;	1
PROKKA_	Phosphoma	VF0392;	O-antigen;	1
PROKKA_	hypothetic	VF0334;	HSI-I;	1
PROKKA_	Histidine t	VF0272;V	FbpABC;F	9
PROKKA_	Chemotaxi	VF0430;V	Flagella;Fl	6
PROKKA_	UDP-4-am	VF0033;V	LPS;LPS;I	3
PROKKA_	Transcripti	VF0222;	S fimbriae;	1
PROKKA_	ATP-deper	VF0074;	ClpP;	1
PROKKA_	Surface pr	VF0116;V	TTSS(SPI-	3
PROKKA_	Superoxide	VF0109;	SodCI;	1
PROKKA_	Glucose-1-	VF0392;	O-antigen;	1

setB

Prot	Info	VF_terms	VF_Names	VF_count
PROKKA_	Isochorism	VF0228;	Enterobact	1
PROKKA_	Flagellar b	VF0474;V	Lateral flag	6
PROKKA_	Spermidine	VF0268;	HitABC;	1
PROKKA_	Flagellar h	VF0394;V	Flagella;La	2
PROKKA_	RNA polyr	VF0430;V	Flagella;Fl	4
PROKKA_	Transcripti	VF0082;V	Type IV pi	3
PROKKA_	Glucose-1-	VF0171;	LPS;	1
PROKKA_	Urease sub	VF0050;	Urease;	1
PROKKA_	putative ox	VF0033;	LPS;	1
PROKKA_	3-oxoacyl-	VF0473;V	Polar flage	2
PROKKA_	Chemotaxi	VF0430;V	Flagella;Fl	2

PROKKA_	Heme/hem	VF0269;	HxuABC;	1
PROKKA_	UDP-gluco	VF0003;V	Capsule;Ca	2
PROKKA_	Putative 2-	VF0268;V	HitABC;Fl	2
PROKKA_	Type II sec	VF0084;V	xcp secreti	4
PROKKA_	Glutamine	VF0272;V	FbpABC;Y	6
PROKKA_	Taurine im	VF0095;	Pyochelin;	1
PROKKA_	Invasion pı	VF0116;V	TTSS(SPI-	20
PROKKA_	Iron(3+)-h	VF0467;V	Acinetobac	2
PROKKA_	Multidrug	VF0451;	MtrCDE;	1
PROKKA_	Transcripti	VF0298;V	MprAB;Ph	5
PROKKA_	UTP--gluc	VF0244;V	Hyaluronic	2
PROKKA_	Lipoprotei	VF0272;V	FbpABC;F	15
PROKKA_	Flagellar b	VF0519;V	Flagella;Fl	15
PROKKA_	Phosphohe	VF0436;V	Capsule I;(	2
PROKKA_	Chemotaxi	VF0430;V	Flagella;Fl	2
PROKKA_	Multidrug	VF0451;	MtrCDE;	1
PROKKA_	Salmonella	VF0116;	TTSS(SPI-	1
PROKKA_	Glucose-1-	VF0436;V	Capsule I;I	2
PROKKA_	Methyl-acc	VF0127;	ACF;	1
PROKKA_	General se	VF0478;	Exe T2SS;	1
PROKKA_	Flagellar L	VF0474;V	Lateral flag	6
PROKKA_	ATP-deper	VF0072;	ClpC;	1
PROKKA_	Spermidine	VF0268;	HitABC;	1
PROKKA_	Flagellar h	VF0474;V	Lateral flag	3
PROKKA_	ABC trans	VF0256;V	Shu;Chu;Y	4
PROKKA_	Twitching	VF0082;V	Type IV pi	2
PROKKA_	UDP-2-ace	VF0033;V	LPS;LPS;(	4
PROKKA_	Major exp	VF0334;	HSI-I;	1
PROKKA_	Multidrug	VF0237;	Ibes;	1
PROKKA_	Dimodular	VF0228;	Enterobact	1
PROKKA_	Transcripti	VF0116;	TTSS(SPI-	1
PROKKA_	Macrolide	VF0451;	MtrCDE;	1
PROKKA_	Oxygen-re	VF0116;V	TTSS(SPI-	2
PROKKA_	Flagellar M	VF0430;V	Flagella;Fl	9
PROKKA_	flagellar as	VF0474;	Lateral flag	1
PROKKA_	Flagellar b	VF0474;V	Lateral flag	8
PROKKA_	Outer mem	VF0105;V	Lpf;Haema	16
PROKKA_	Flagellin	VF0473;V	Polar flage	2
PROKKA_	Glucose-6-	VF0465;V	Capsule;LI	2
PROKKA_	Chemotaxi	VF0430;V	Flagella;Fl	3
PROKKA_	D-inositol-	VF0367;	LPS;	1
PROKKA_	Flagellum	VF0519;	Flagella;	1
PROKKA_	Flagellar L	VF0394;V	Flagella;La	6
PROKKA_	Transcripti	VF0091;V	Alginate;B	2
PROKKA_	3-oxoacyl-	VF0436;V	Capsule I;Y	3
PROKKA_	Major oute	VF0081;	Porin;	1
PROKKA_	Response r	VF0428;	Bsa T3SS;	1
PROKKA_	hypothetic	VF0334;	HSI-I;	1
PROKKA_	Competenc	VF0082;V	Type IV pi	2
PROKKA_	Levodione	VF0473;V	Polar flage	2
PROKKA_	putative m	VF0136;V	Yersiniaba	2
PROKKA_	dTDP-4-de	VF0171;	LPS;	1
PROKKA_	Iron impor	VF0136;	Yersiniaba	1
PROKKA_	Response r	VF0262;V	LetA/S;Ty	6
PROKKA_	flagellar bi	VF0474;V	Lateral flag	7
PROKKA_	Motility pr	VF0157;V	Flagella;Po	6
PROKKA_	4-carboxy-	VF0033;	LPS;	1
PROKKA_	flagellar ba	VF0273;V	Flagella;Fl	2

PROKKA_	Transcripti	VF0317;V	DevRS;Bsa	6
PROKKA_	Putative ph	VF0268;V	HitABC;A	6
PROKKA_	Motility pr	VF0430;V	Flagella;La	3
PROKKA_	UDP-N-ac	VF0465;V	Capsule;Ca	2
PROKKA_	RNA polyı	VF0157;V	Flagella;Fl	6
PROKKA_	Inositol ph	VF0116;	TTSS(SPI-	1
PROKKA_	High-affini	VF0268;V	HitABC;A	10
PROKKA_	Transcripti	VF0430;	Flagella;	1
PROKKA_	Peptide me	VF0456;	MsrAB;	1
PROKKA_	Cell invasi	VF0116;	TTSS(SPI-	1
PROKKA_	Flagellar b	VF0116;V	TTSS(SPI-	18
PROKKA_	Flagellar m	VF0430;V	Flagella;Fl	6
PROKKA_	Flagellar h	VF0273;V	Flagella;Fl	3
PROKKA_	hypothetica	VF0334;V	HSI-I;T6SS	2
PROKKA_	Enoyl-[acy	VF0228;	Enterobact	1
PROKKA_	Basal-body	VF0430;V	Flagella;Fl	2
PROKKA_	Major oute	VF0081;	Porin;	1
PROKKA_	Flagellar m	VF0474;V	Lateral flag	2
PROKKA_	ATP synth	VF0035;	TTSS;	1
PROKKA_	C4-dicarbo	VF0082;V	Type IV pi	5
PROKKA_	Nitrogen re	VF0473;	Polar flage	1
PROKKA_	putative ox	VF0228;V	Enterobact	2
PROKKA_	Putative gl	VF0078;	LOS;	1
PROKKA_	Laminin-bi	VF0221;V	Type 1 fim	16
PROKKA_	Cholix tox	VF0086;	ExoA;	1
PROKKA_	Chaperone	VF0334;V	HSI-I;T6SS	2
PROKKA_	Putative m	VF0225;V	Hemolysin	4
PROKKA_	Phenyloxa	VF0094;	Pyoverdine	1
PROKKA_	ABC trans	VF0272;V	FbpABC;C	2
PROKKA_	Chaperone	VF0334;V	HSI-I;T6SS	3
PROKKA_	Flagellar b	VF0430;V	Flagella;Fl	11
PROKKA_	Flagellar b	VF0474;V	Lateral flag	13
PROKKA_	CDP-gluco	VF0392;V	O-antigen;	2
PROKKA_	Flagella ba	VF0519;	Flagella;	1
PROKKA_	2-hydroxyı	VF0089;	Rhamnolip	1
PROKKA_	L-ornithine	VF0094;V	Pyoverdine	3
PROKKA_	D-methion	VF0513;	IlpA;	1
PROKKA_	Phosphogl	VF0465;V	Capsule;O-	2
PROKKA_	Superoxide	VF0169;V	SodB;SodA	2
PROKKA_	Flagellar b	VF0430;V	Flagella;Fl	8
PROKKA_	Transcripti	VF0262;V	LetA/S;Ty	2
PROKKA_	UDP-N-ac	VF0033;	LPS;	1
PROKKA_	ADP-L-gly	VF0044;V	LOS;LOS;	3
PROKKA_	hypothetica	VF0334;V	HSI-I;T6SS	3
PROKKA_	Chemotaxi	VF0157;V	Flagella;Fl	6
PROKKA_	Flagellar b	VF0430;V	Flagella;Fl	15
PROKKA_	Flagellar h	VF0273;V	Flagella;Fl	5
PROKKA_	Hemin tran	VF0234;V	Chu;Chu;S	3
PROKKA_	Undecapre	VF0124;	LPS;	1
PROKKA_	Efflux pum	VF0451;	MtrCDE;	1
PROKKA_	Intracellula	VF0334;	HSI-I;	1
PROKKA_	Putative m	VF0430;	Flagella;	1
PROKKA_	3-deoxy-m	VF0473;	Polar flage	1
PROKKA_	Sulfate/thi	VF0268;	HitABC;	1
PROKKA_	Arabinose	VF0095;	Pyochelin;	1
PROKKA_	Transcripti	VF0262;V	LetA/S;Ty	4
PROKKA_	3-oxoacyl-	VF0473;V	Polar flage	2
PROKKA_	Alpha-D-ri	VF0272;V	FbpABC;Y	2

PROKKA_	N,N'-diace	VF0171;V	LPS;Capsu	7
PROKKA_	Transcripti	VF0091;	Alginate;	1
PROKKA_	Phosphate	VF0228;V	Enterobact	14
PROKKA_	Flagellar b	VF0473;V	Polar flage	2
PROKKA_	Respirator	VF0302;	Nitrate red	1
PROKKA_	hypothetic	VF0334;V	HSI-I;T6S	2
PROKKA_	putative A	VF0136;	Yersiniaba	1
PROKKA_	Transcripti	VF0298;V	MprAB;Bv	4
PROKKA_	Flagellin E	VF0473;V	Polar flage	4
PROKKA_	Carnitine c	VF0414;	RicA;	1
PROKKA_	Malonyl C	VF0136;V	Yersiniaba	2
PROKKA_	GDP-manr	VF0392;V	O-antigen;	6
PROKKA_	60 kDa cha	VF0159;	Hsp60;	1
PROKKA_	Chaperone	VF0486;V	Type I pili	9
PROKKA_	Chemotaxi	VF0430;V	Flagella;Fl	4
PROKKA_	Sensory tra	VF0298;V	MprAB;Bv	4
PROKKA_	hypothetic	VF0429;	T6SS-1;	1
PROKKA_	Methyl-acc	VF0430;	Flagella;	1
PROKKA_	Flagellum-	VF0474;V	Lateral fla	17
PROKKA_	Multidrug	VF0450;	FarAB;	1
PROKKA_	Colicin I re	VF0228;V	Enterobact	3
PROKKA_	Lipopolysa	VF0078;V	LOS;LPS;	4
PROKKA_	Salicylate	VF0095;	Pyochelin;	1
PROKKA_	chemotaxis	VF0430;	Flagella;	1
PROKKA_	Chaperone	VF0116;	TTSS(SPI-	1
PROKKA_	Motility pr	VF0430;V	Flagella;Fl	3
PROKKA_	O-GlcNAc	VF0389;	mu-toxin;	1
PROKKA_	Chaperone	VF0072;V	ClpC;HSI-	3
PROKKA_	Yqey-like	VF0156;	Dot/Icm;	1
PROKKA_	Arabinose	VF0082;	Type IV pi	1
PROKKA_	DNA repai	VF0457;	RecN;	1
PROKKA_	Ditrans,po	VF0361;	Capsule;	1
PROKKA_	Multidrug	VF0451;V	MtrCDE;Il	2
PROKKA_	Sigma fact	VF0091;	Alginate;	1
PROKKA_	Hemin trar	VF0467;	Acinetobac	1
PROKKA_	Iron impor	VF0136;V	Yersiniaba	2
PROKKA_	Response r	VF0082;	Type IV pi	1
PROKKA_	Chemotaxi	VF0474;V	Lateral fla	3
PROKKA_	Transcripti	VF0368;V	BvrR-BvrS	4
PROKKA_	Sulfate trar	VF0272;	FbpABC;	1
PROKKA_	hypothetic	VF0429;V	T6SS-1;T6	2
PROKKA_	Type-1 fim	VF0031;V	Fimbriae;F	14
PROKKA_	Twitching	VF0082;V	Type IV pi	6
PROKKA_	Flagellar m	VF0474;	Lateral fla	1
PROKKA_	Type II sec	VF0084;V	xcp secreti	11
PROKKA_	Linear grai	VF0299;	Mycobacti	1
PROKKA_	D-alanyl-D	VF0468;	PbpG;	1
PROKKA_	Flagellin	VF0473;V	Polar flage	2
PROKKA_	hypothetic	VF0430;V	Flagella;Fl	12
PROKKA_	Hemin imp	VF0227;V	Chu;Shu;E	5
PROKKA_	Flagellar h	VF0430;V	Flagella;Fl	8
PROKKA_	Swarming	VF0298;V	MprAB;Ph	4
PROKKA_	RNA polyr	VF0112;V	RpoS;RpoS	3
PROKKA_	Flagellar P	VF0474;V	Lateral fla	8
PROKKA_	D-beta-D-h	VF0044;V	LOS;LOS;	2
PROKKA_	bacteriopha	VF0475;	Tap type I	1
PROKKA_	2,3-dihydro	VF0095;V	Pyochelin;	8
PROKKA_	8-amino-7-	VF0436;	Capsule I;	1

PROKKA_	Flagellar P	VF0430;V	Flagella;Fl	8
PROKKA_	putative A	VF0225;V	Hemolysin	2
PROKKA_	Methyl-acc	VF0430;	Flagella;	1
PROKKA_	Alginate bi	VF0436;V	Capsule I;A	5
PROKKA_	Yop protei	VF0116;	TTSS(SPI-	1
PROKKA_	Catalase pı	VF0454;	KatA;	1
PROKKA_	Type II sec	VF0082;V	Type IV pi	5
PROKKA_	N-acylneuı	VF0465;V	Capsule;Ca	6
PROKKA_	Alpha-D-k	VF0033;V	LPS;Capsu	2
PROKKA_	Chaperone	VF0036;V	Haemagglu	8
PROKKA_	hypothetic	VF0334;	HSI-I;	1
PROKKA_	Flagellar b	VF0430;V	Flagella;Fl	20
PROKKA_	Transcripti	VF0091;	Alginate;	1
PROKKA_	Flagellar h	VF0474;V	Lateral flag	8
PROKKA_	Imidazole g	VF0171;	LPS;	1
PROKKA_	Flagellum-	VF0430;V	Flagella;Fl	15
PROKKA_	hypothetic	VF0334;	HSI-I;	1
PROKKA_	Lipid A ex	VF0225;V	Hemolysin	4
PROKKA_	Biofilm op	VF0014;	Intercellula	1
PROKKA_	colanic aci	VF0003;	Capsule;	1
PROKKA_	Transcripti	VF0519;V	Flagella;Fl	4
PROKKA_	Transcripti	VF0368;V	BvrR-BvrS	4
PROKKA_	Fimbrial as	VF0082;V	Type IV pi	2
PROKKA_	Di-/tripept	VF0293;	IraAB;	1
PROKKA_	D-beta-hyd	VF0473;V	Polar flage	3
PROKKA_	Putative ty	VF0154;	<i>lsp</i>	1
PROKKA_	hypothetic	VF0334;V	HSI-I;T6S	2
PROKKA_	TonB-depe	VF0269;V	HxuABC;C	4
PROKKA_	putative sic	VF0467;V	Acinetobac	3
PROKKA_	Respirator	VF0302;	Nitrate red	1
PROKKA_	L-lysine N	VF0229;V	Aerobactin	3
PROKKA_	Phosphate	VF0298;V	MprAB;Ph	4
PROKKA_	Lipoprotei	VF0272;V	FbpABC;H	7
PROKKA_	Carbonic a	VF0396;	Mig-5;	1
PROKKA_	hypothetic	VF0429;V	T6SS-1;T6	2
PROKKA_	putative A	VF0268;V	HitABC;Fl	11
PROKKA_	UDP-N-ac	VF0274;	Capsule;	1
PROKKA_	Type III se	VF0118;V	TTSS;Bsa	2
PROKKA_	Chemotaxi	VF0430;V	Flagella;Fl	3
PROKKA_	Outer mem	VF0224;V	F1C fimbri	10
PROKKA_	UDP-4-am	VF0465;V	Capsule;LI	2
PROKKA_	dTDP-gluc	VF0171;V	LPS;Capsu	9
PROKKA_	Chaperone	VF0031;V	Fimbriae;T	9
PROKKA_	Cell divisi	VF0268;V	HitABC;C	20
PROKKA_	UDP-4-am	VF0033;V	LPS;Capsu	5
PROKKA_	High-affini	VF0228;V	Enterobact	9
PROKKA_	Respirator	VF0302;	Nitrate red	1
PROKKA_	Urease sub	VF0050;	Urease;	1
PROKKA_	Hemin-bin	VF0256;V	Shu;Chu;C	3
PROKKA_	Outer mem	VF0451;V	MtrCDE;Ib	2
PROKKA_	Flagellar h	VF0474;V	Lateral flag	2
PROKKA_	Transcripti	VF0298;V	MprAB;Bv	5
PROKKA_	Cobalt-zin	VF0451;	MtrCDE;	1
PROKKA_	Outer mem	VF0451;V	MtrCDE;Ib	2
PROKKA_	Lipoprotei	VF0116;V	TTSS(SPI-	3
PROKKA_	Oxygen reg	VF0262;V	LetA/S;De	2
PROKKA_	Outer mem	VF0221;V	Type 1 fim	9
PROKKA_	Virulence	VF0111;V	PhoPQ;Bv	4

PROKKA_	Transcripti	VF0111;V	PhoPQ;Mp	6
PROKKA_	Outer mem	VF0486;V	Type I pili;	18
PROKKA_	CDP-parat	VF0171;V	LPS;Capsu	11
PROKKA_	Putative ty	VF0154;V	<i>lsp</i>	3
PROKKA_	ECF RNA	VF0296;V	SigH;SigE	3
PROKKA_	Transcripti	VF0298;V	MprAB;Ph	5
PROKKA_	3-oxoacyl-	VF0473;V	Polar flage	3
PROKKA_	Octopine p	VF0268;V	HitABC;Fl	10
PROKKA_	Putative gl	VF0078;V	LOS;LOS;	2
PROKKA_	hypothetic	VF0334;V	HSI-I;T6S	3
PROKKA_	Pseudolysi	VF0087;	LasB;	1
PROKKA_	Lipopolysa	VF0268;V	HitABC;Fl	11
PROKKA_	Imidazole	VF0171;	LPS;	1
PROKKA_	Glucose 1-	VF0473;V	Polar flage	3
PROKKA_	Transcripti	VF0262;V	LetA/S;Ty	5
PROKKA_	Vitamin B	VF0234;V	Chu;Shu;	2
PROKKA_	Spermidin	VF0268;	HitABC;	1
PROKKA_	N,N'-diace	VF0171;V	LPS;Capsu	5
PROKKA_	Major oute	VF0081;	Porin;	1
PROKKA_	Guanosine	VF0287;V	RelA;RelA	2
PROKKA_	hypothetic	VF0334;	HSI-I;	1
PROKKA_	hypothetic	VF0429;V	T6SS-1;T6	2
PROKKA_	Motility pr	VF0430;V	Flagella;Fl	4
PROKKA_	putative pe	VF0455;V	MntABC;F	5
PROKKA_	Outer mem	VF0116;V	TTSS(SPI-	6
PROKKA_	Type II sec	VF0084;V	xcp secreti	3
PROKKA_	Chemotaxi	VF0262;	LetA/S;	1
PROKKA_	Glycerol-3	VF0264;	Hpt;	1
PROKKA_	Multidrug	VF0451;	MtrCDE;	1
PROKKA_	Cell invasi	VF0116;V	TTSS(SPI-	3
PROKKA_	3-oxoacyl-	VF0473;V	Polar flage	2
PROKKA_	hypothetic	VF0334;V	HSI-I;T6S	3
PROKKA_	Flagellin	VF0473;V	Polar flage	2
PROKKA_	Type IV pi	VF0082;	Type IV pi	1
PROKKA_	Outer mem	VF0451;V	MtrCDE;Il	2
PROKKA_	3-oxoacyl-	VF0473;V	Polar flage	2
PROKKA_	p-hydroxyl	VF0450;V	FarAB;Do	2
PROKKA_	NAD-depe	VF0444;	Lap;	1
PROKKA_	Chemotaxi	VF0519;	Flagella;	1
PROKKA_	3-oxoacyl-	VF0473;V	Polar flage	2
PROKKA_	Catalase-p	VF0168;V	KatAB;Ka	3
PROKKA_	Flagellar b	VF0157;V	Flagella;Fl	10
PROKKA_	Glucose-6-	VF0465;	Capsule;	1
PROKKA_	HTH-type	VF0362;	BopD;	1
PROKKA_	Type 4 pre	VF0478;V	Exe T2SS;	3
PROKKA_	Glucose 1-	VF0473;V	Polar flage	2
PROKKA_	flagellar bi	VF0116;V	TTSS(SPI-	4
PROKKA_	GDP-L-fuc	VF0392;V	O-antigen;	5
PROKKA_	putative bi	VF0268;V	HitABC;Fl	2
PROKKA_	High-affini	VF0272;V	FbpABC;A	11
PROKKA_	UDP-4-am	VF0033;V	LPS;Capsu	5
PROKKA_	Bifunction	VF0323;V	Capsule;Ca	3
PROKKA_	Multidrug	VF0451;	MtrCDE;	1
PROKKA_	Manganese	VF0455;	MntABC;	1
PROKKA_	hypothetic	VF0334;	HSI-I;	1
PROKKA_	Transglyco	VF0116;	TTSS(SPI-	1
PROKKA_	Phthiocero	VF0309;V	PDIM;Cap	2
PROKKA_	UDP-N-ac	VF0091;	Alginate;	1

PROKKA_	Chemotaxi	VF0519;	Flagella;	1
PROKKA_	putative ox	VF0228;V	Enterobact	2
PROKKA_	Peptidogly	VF0430;V	Flagella;Fl	3
PROKKA_	Flagellin	VF0473;V	Polar flage	2
PROKKA_	Isochorism	VF0228;	Enterobact	1
PROKKA_	Flagellar m	VF0430;V	Flagella;Fl	8
PROKKA_	putative sid	VF0361;	Capsule;	1
PROKKA_	hypothetica	VF0334;V	HSI-I;T6SS	3
PROKKA_	UDP-2-ace	VF0033;	LPS;	1
PROKKA_	UDP-3-O-[	VF0436;	Capsule I;	1
PROKKA_	dTDP-4-de	VF0171;V	LPS;O-ant	2
PROKKA_	Non-hemol	VF0470;V	Phospholip	2
PROKKA_	Histidinol-	VF0323;V	Capsule;Ca	2
PROKKA_	Type II sec	VF0155;V	Type IV pi	10
PROKKA_	3-oxoacyl-	VF0473;V	Polar flage	3
PROKKA_	Chemotaxi	VF0474;	Lateral flag	1
PROKKA_	Flagellar b	VF0430;V	Flagella;Fl	15
PROKKA_	RNA polym	VF0112;V	RpoS;SigA	2
PROKKA_	Type-1 fim	VF0105;V	Lpf;Type 1	13
PROKKA_	Long-chain	VF0095;V	Pyochelin;	8
PROKKA_	UDP-2,4-d	VF0465;V	Capsule;Po	2
PROKKA_	Urease acc	VF0050;	Urease;	1
PROKKA_	Methylisoc	VF0253;	Isocitrate l	1
PROKKA_	Chaperone	VF0031;V	Fimbriae;H	10
PROKKA_	Alginate bi	VF0082;V	Type IV pi	4
PROKKA_	Flagellar b	VF0474;V	Lateral flag	19
PROKKA_	Dimodular	VF0228;V	Enterobact	2
PROKKA_	Aldehyde-a	VF0444;	Lap;	1
PROKKA_	Outer mem	VF0105;V	Lpf;F1C fi	18
PROKKA_	Poly-beta-1	VF0014;	Intercellula	1
PROKKA_	Alcohol de	VF0444;	Lap;	1
PROKKA_	Flagellar b	VF0394;V	Flagella;Fl	3
PROKKA_	hypothetica	VF0334;	HSI-I;	1
PROKKA_	Organic hy	VF0222;	S fimbriae;	1
PROKKA_	Twitching	VF0475;V	Tap type IV	3
PROKKA_	Nitrogen re	VF0082;	Type IV pi	1
PROKKA_	Flagellar b	VF0474;V	Lateral flag	14
PROKKA_	putative AT	VF0116;V	TTSS(SPI-	17
PROKKA_	Chaperone	VF0334;V	HSI-I;T6SS	3
PROKKA_	Flagellar h	VF0394;V	Flagella;Fl	2
PROKKA_	60 kDa cha	VF0159;	Hsp60;	1
PROKKA_	6-hydroxyn	VF0100;	Pyocyanin;	1
PROKKA_	Chemotaxi	VF0430;	Flagella;	1
PROKKA_	Dimodular	VF0467;	Acinetobac	1
PROKKA_	Type-1 fim	VF0105;V	Lpf;Type 1	12
PROKKA_	putative ef	VF0451;V	MtrCDE;Ib	2
PROKKA_	HrpJ-like d	VF0118;	TTSS;	1
PROKKA_	Flagellar b	VF0519;	Flagella;	1
PROKKA_	Multidrug	VF0028;V	Cya;Hemo	3
PROKKA_	Long-chain	VF0095;V	Pyochelin;	8
PROKKA_	Chemotaxi	VF0430;V	Flagella;Fl	2
PROKKA_	Efflux pum	VF0451;	MtrCDE;	1
PROKKA_	Sensor pro	VF0082;	Type IV pi	1
PROKKA_	Arabinose	VF0323;	Capsule;	1
PROKKA_	Transcripti	VF0298;V	MprAB;Bv	4
PROKKA_	Putative be	VF0163;V	enh loci;en	2
PROKKA_	Hemin tran	VF0256;V	Shu;Chu;C	4
PROKKA_	hypothetica	VF0105;	Lpf;	1

PROKKA_	Histidine t	VF0272;V	FbpABC;F	13
PROKKA_	Flagellar b	VF0430;V	Flagella;Fl	6
PROKKA_	Multidrug	VF0451;	MtrCDE;	1
PROKKA_	putative To	VF0094;V	Pyoverdine	2
PROKKA_	Lipopolysa	VF0085;V	LPS;LOS;	2
PROKKA_	Flagellar h	VF0273;V	Flagella;Fl	5
PROKKA_	Antibiotic	VF0237;	Ibes;	1
PROKKA_	Sulfate tra	VF0268;	HitABC;	1
PROKKA_	FK506-bin	VF0153;	Mip;	1
PROKKA_	Long-chair	VF0095;V	Pyochelin;	12
PROKKA_	Sorbitol de	VF0473;V	Polar flage	3
PROKKA_	2-C-methy	VF0043;	Capsule;	1
PROKKA_	Ferrichrom	VF0467;	Acinetobac	1
PROKKA_	Alginate bi	VF0091;V	Alginate;C	5
PROKKA_	Acetoacety	VF0473;V	Polar flage	3
PROKKA_	flagellar ba	VF0519;	Flagella;	1
PROKKA_	flagellar ba	VF0519;V	Flagella;Fl	2
PROKKA_	Methyl-acc	VF0126;	TCP;	1
PROKKA_	Ferric upta	VF0113;	Fur;	1
PROKKA_	Urease sub	VF0050;	Urease;	1
PROKKA_	Outer mem	VF0221;V	Type 1 fim	17
PROKKA_	Galactose/	VF0268;V	HitABC;Fl	3
PROKKA_	Isocitrate l	VF0253;	Isocitrate l	1
PROKKA_	Transcripti	VF0082;V	Type IV pi	5
PROKKA_	Transcripti	VF0262;V	LetA/S;Bv	3
PROKKA_	Phosphohe	VF0326;V	LOS;Capsu	3
PROKKA_	2,3-dihydro	VF0228;V	Enterobact	3
PROKKA_	Surface pro	VF0428;V	Bsa T3SS;	6
PROKKA_	UDP-N-ac	VF0144;V	Capsule;Ca	4
PROKKA_	Outer mem	VF0322;	CadF;	1
PROKKA_	HTH-type	VF0362;	BopD;	1
PROKKA_	3-deoxy-D	VF0085;V	LPS;LOS;	2
PROKKA_	Magnesiun	VF0106;	MgtBC;	1
PROKKA_	CDP-abequ	VF0392;V	O-antigen;	2
PROKKA_	Ribosomal	VF0156;	Dot/Icm;	1
PROKKA_	Flagellar h	VF0474;	Lateral flag	1
PROKKA_	Trifunction	VF0422;	AdsA;	1
PROKKA_	Fe(3+) ion	VF0272;	FbpABC;	1
PROKKA_	Methionine	VF0268;V	HitABC;C	9
PROKKA_	Lipopolysa	VF0272;V	FbpABC;F	6
PROKKA_	flagellar bi	VF0116;V	TTSS(SPI-	4
PROKKA_	Lipid A bio	VF0466;V	LPS;LOS;	2
PROKKA_	Outer mem	VF0235;V	S fimbriae;	19
PROKKA_	Flagellar b	VF0519;V	Flagella;Fl	6
PROKKA_	ECF RNA	VF0091;V	Alginate;S	2
PROKKA_	Flagellar M	VF0474;V	Lateral flag	7
PROKKA_	Endonucle	VF0422;	AdsA;	1
PROKKA_	Pilus assen	VF0082;V	Type IV pi	2
PROKKA_	putative ma	VF0082;	Type IV pi	1
PROKKA_	Linear gra	VF0136;	Yersiniaba	1
PROKKA_	KDP opero	VF0298;V	MprAB;Ph	4
PROKKA_	Hemin tran	VF0234;V	Chu;Shu;C	5
PROKKA_	3-oxoacyl-	VF0436;	Capsule I;	1
PROKKA_	ADP-hepto	VF0078;V	LOS;LPS;l	4
PROKKA_	GTP pyrop	VF0260;V	RelA;RelA	2
PROKKA_	Chemotaxi	VF0430;V	Flagella;Fl	3
PROKKA_	Quinone o	VF0309;	PDIM;	1
PROKKA_	Dimodular	VF0094;	Pyoverdine	1

PROKKA_	Colicin I re	VF0048;V	HmbR;Hpu	6
PROKKA_	Type II sec	VF0084;	xcp secreti	1
PROKKA_	Type III se	VF0116;V	TTSS(SPI-	2
PROKKA_	Lipopolysa	VF0085;	LPS;	1
PROKKA_	Ferrous iro	VF0160;	FeoAB;	1
PROKKA_	Phosphoma	VF0392;V	O-antigen;	2
PROKKA_	hypothetica	VF0334;	HSI-I;	1
PROKKA_	Histidine t	VF0272;V	FbpABC;H	9
PROKKA_	Chemotaxi	VF0430;V	Flagella;Fl	10
PROKKA_	UDP-4-am	VF0465;V	Capsule;LI	5
PROKKA_	Transcripti	VF0222;	S fimbriae;	1
PROKKA_	ATP-deper	VF0074;	ClpP;	1
PROKKA_	Surface pro	VF0116;V	TTSS(SPI-	3
PROKKA_	Superoxide	VF0109;	SodCI;	1
PROKKA_	Glucose-1-	VF0392;	O-antigen;	1
